# The Influence of Family Caregiver Knowledge and Behavior on Elderly Diabetic Patients’ Quality of Life in Northern Thailand

**DOI:** 10.3390/ijerph191610216

**Published:** 2022-08-17

**Authors:** Kitbordin Thongduang, Waraporn Boonchieng, Sineenart Chautrakarn, Parichat Ong-Artborirak

**Affiliations:** 1 Faculty of Public Health, Chiang Mai University, Chiang Mai 50200, Thailand; 2 Center of Excellence in Community Health Informatics, Chiang Mai University, Chiang Mai 50200, Thailand

**Keywords:** quality of life, diabetes mellitus, knowledge, behavior, caregiver, elderly

## Abstract

The quality of life (QoL) of elderly diabetic patients may be affected by caregiver factors, but this has received little empirical support. The objective of this cross-sectional study is to determine the influence of family caregivers’ diabetes knowledge and behavior on the QoL among elderly patients with diabetes mellitus (DM). The participants included 354 elderly patients with Type 2 DM and their family caregivers, who were recruited through multistage sampling from five districts in Chiang Mai, Thailand. Face-to-face interviews with DM patients were conducted using the Thai Simplified Diabetes Knowledge Scale (T-SDKS), the Thai version of the Diabetes Self-Management Questionnaire (DSMQ) for self-care behaviors, and the Thai version of the World Health Organization Quality of Life for Older People (WHOQOL-OLD) scale. For caregivers, their diabetes knowledge was measured by T-SDKS and patient-care or supportive behaviors were developed based on DSMQ. The results showed a moderate level of QoL among elderly diabetic patients. According to simple linear regression analysis, the QoL score among elderly DM patients was positively associated with their diabetes knowledge (B = 1.25), self-care behaviors (B = 3.00), caregivers’ knowledge (B = 0.97), and supportive behavior from caregivers (B = 2.92) at a significance level of *p* < 0.01. In the multivariable model, patients’ self-care behaviors (B = 1.58, *p* = 0.001), caregivers’ knowledge (B = 0.58, *p* = 0.001), and patient-care behaviors (B = 1.38, *p* = 0.004) were significantly associated with QoL among DM patients when controlling for patient factors, including age, body mass index (BMI), education, and living arrangements, which accounted for 27.0% of the variance. This indicates that caregivers’ adequate diabetes knowledge and appropriate supportive behaviors may impact the QoL of elderly diabetic patients. Health care providers should prioritize motivating and empowering family caregivers to pay more attention to the patient for the success goal.

## 1. Introduction

Diabetes mellitus (DM) remains a global problem with increasing cases, particularly among the elderly. Physical degradation in elderly people with DM can lead to an increased risk of complications, including death and diminished quality of life (QoL) [1,2,3,4,5]. The International Diabetes Federation estimates that by 2045, there will be 783.2 million people with diabetes worldwide, with the majority being people ages 75–79 years [6]. In Thailand, the 6th National Health Examination Survey in 2019–2020 revealed the prevalence of diabetes was 9.5%, which was higher than the data from the previous report, with the largest increase being among people ages 60–69 years [7]. Furthermore, diabetes incidence and mortality rates have increased in the northern area of Thailand, with the highest rates in Chiang Mai province [8]. Diabetes therapy necessitates self-care or self-management, which should focus on improving the overall health and well-being of diabetics [9,10]

QoL is a good indicator of diabetes management. Higher QoL has been linked to good diabetes outcomes and patients’ glycemic control [11,12]. Self-care behaviors are a crucial component of diabetes management [4,11]. Self-care behaviors require appropriate patient knowledge and encouragement [13]. Improved diabetes knowledge and self-care behaviors enable patients to improve their lifestyle and lower the risk of diabetes-related complications [14]. Previous studies found a relationship between diabetes knowledge and self-care behaviors [15,16]. Additionally, a correlation between knowledge and QoL of elderly patients with DM has been observed [17], and self-care behaviors have been associated with their QoL [16,18,19]. In addition to these patient features, social or family support may have an influence on health.

Family members are crucial in providing care for elderly patients. Both the patient and family play an important role in the maintenance of lifestyle changes and the management of diabetes [20,21]. Assistance with performing daily activities, accessing health services, providing financial support, managing diet, taking medications, and checking blood glucose are examples of family support [20,21]. In a previous study, patients who received increased social support were more prepared to execute newly learned behaviors and ways of thinking [22]. This demonstrates how having a family caregiver is beneficial for the health promotion of older diabetic patients and may enhance the patients’ QoL. As a result, this study hypothesizes that family caregivers’ diabetes knowledge and behaviors would have an influence on the QoL of their elderly DM family members. A few studies have been conducted on how patient–caregiver dyads relate to one another. The main objective of this study was to assess the association of family caregivers’ diabetes knowledge and patient-care behaviors with the QoL of elderly diabetic patients in northern Thailand. As the Thailand National Noncommunicable Disease Plan includes a strategy of development and empowerment of people for health promotion, disease prevention and control, resulting in well-being maintenance. The findings of this study can provide information to help develop policies and health promotion strategies for elderly diabetic patients and can advocate for public health providers to emphasize family and community involvement in support of holistic care for elderly people with diabetes.

## 2. Materials and Methods

### 2.1. Study Design and Subjects

**Figure 1:**
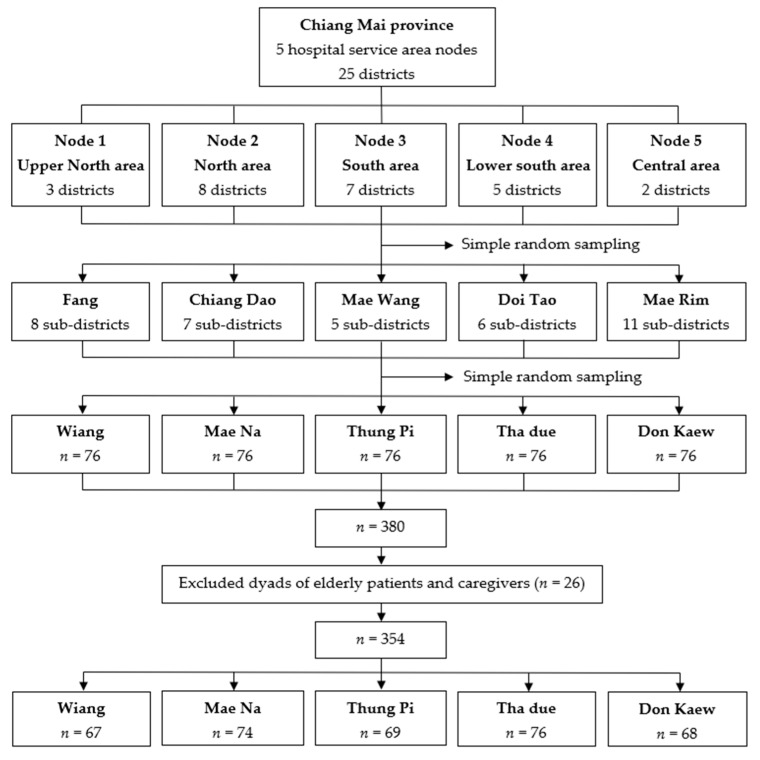
A diagram of the multistage sampling.

This cross-sectional study used a QoL survey conducted among elderly patients with DM and their family caregivers from five different districts (Fang, Chiang Dao, Mae Rim, Doi Lor, and Doi Tao) in Chiang Mai, northern Thailand. We used multistage sampling to pick participants (Figure 1). First, we randomly selected one district from each service area node according to the service plan of the Chiang Mai Provincial Public Health Office. From each district, we chose one sub-district at random. The subjects in each node were then selected using simple random sampling from a list of elderly diabetic patients at the Sub-District Health Promotion Hospital or a community hospital in the area. The inclusion criteria were elderly diabetic patients with a doctor’s diagnosis of DM for more than one year and age ≥ 60 years. The representative subjects were contacted in person at their homes by the research team, which included the researcher and two trained research assistants with an academic level who were located in the research area. Caregivers of diabetic patients who were eligible for participation were approached. The caregiver criteria included being a primary caregiver over the age of 18, having a familial relationship with the patient, caring for the DM patient for more than a year, and not having diabetes. We excluded some dyads of elderly patients and caregivers if they were unwilling to participate in the study or the caregivers did not meet the criteria.

G-power was used to compute the sample size using a correlation bivariate model with an effect size of 0.15, a 95% confidence level, an 80% power, and a two-tailed test. The sample size was increased by 10%, resulting in 380 dyads of participants. The total final sample size of this study was 354 dyads of elderly diabetic patients and their family caregiver. The study was approved by the Committee of Research Ethics, Faculty of Public Health, Chiang Mai University (No. ET002/2022). Written informed consent was obtained from all subjects involved in the study.

### 2.2. Data Collection

The data were collected from January to March 2022. Face-to-face interviews were conducted by the research team using a combined questionnaire specific to elderly people with diabetes and their family caregiver. The interview time for elderly diabetic patients was approximately 30–40 min, and it was at about 15–25 min for caregivers. There were five parts for the elderly person with diabetes. Part 1: Elderly Diabetes Characteristics and Clinical Diabetes Data included sex, age, weight, height, education, marital status, occupation, perceived financial status, smoking, alcohol consumption, living arrangement, DM duration and treatment, comorbidities, and complications. Part 2: Questionnaire for Activities in Daily Living (ADL) using the modified Barthel ADL Index [23], which was translated into Thai with a cut-off point for independent patients [24,25]. It is widely and easily used to determine the ability of the elderly to carry out their daily routines, with 10 questions covering basic activities, such as feeding, bathing, grooming, dressing, movement, walking, going up and down stairs, using the toilet, and defecation. Part 3: Diabetes Knowledge used a 20-item Simplified Diabetes Knowledge Scale (SDKS) [26] translated and verified in Thai (T-SDKS) [27]. It includes questions about diet, risk factors, and self-care. One point is awarded for each correct answer, for a total possible score of 20. A high score indicates high knowledge of diabetes. Part 4: Diabetes Self-Care Behaviors used the 16-item Diabetes Self-Management Questionnaire (DSMQ) [28], which was translated and validated for the Thai language [29]. DSMQ is a four-point Likert scale instrument ranging from 0 (does not apply to me) to 3 (applies to me very much). The sum scale (SS) determines overall self-care, and the four subscales consist of glucose management (GM), dietary control (DC), physical activity (PA), and health-care use (HU). Scale scores were computed by adding item scores and then converting them to a scale of 0 to 10 ((raw score/theoretical maximum score) × 10). Each subscale has a positive and negative item. Higher scores indicate more effective self-care behaviors. Part 5: Quality of Life (QoL) used the 24-item World Health Organization Quality of Life in Older People (WHOQOL-OLD) scale [30] translated into the Thai language [31]. Responses used a five-point Likert scale ranging from 1 to 5. It consists of six facets: sensory ability (SAB), autonomy (AUT), social participation (SOP), physical function or past-present-future activity (PPF), death and dying (DAD), and intimacy (INT). Possible scores range from 24 to 120, with 24–57 indicating poor QoL, 58–98 indicating moderate QoL, and 99–120 indicating good QoL.

The questionnaire for the family caregiver was divided into three parts. Part 1: Caregiver Characteristics and Care Information included sex, age, weight, height, education, marital status, family income, smoking, alcohol consumption, relationship to patient, years of caring experience, time spent caring for the individual each day, and secondary caregiver. Part 2: Diabetes Knowledge used the same 20-item T-SDKS [27] as for the elderly diabetes participants. Part 3: Patient-Care Behaviors or Supportive Behaviors for Elderly Diabetes was developed based on the 16-item DSMQ [29]. Examples of questions were “I encourage elderly diabetic patients to choose the simplest foods to achieve optimal blood sugar levels” and “Regarding patient’s diabetes care, I should take the patient to see his/her medical practitioner(s) more often.” Similarly, it was interpreted according to the DSMQ used for the diabetic patients. Part 3 was reviewed and validated by a panel of three content experts consisting of an endocrine physician, a family nurse, and an expert in family health research. All the instruments were pilot tested with 30 patient–caregiver dyads who lived in a nearby area and had the same characteristics as the participants in this study. Regarding the reliability, the Kuder–Richardson (KR-20) coefficient was 0.73 for patients’ diabetes knowledge and 0.73 for caregivers’ diabetes knowledge. Cronbach’s alpha coefficients were 0.71, 0.87, 0.71, and 0.76 for ADL, self-care behaviors, QoL, and patient-care behaviors, respectively.

### 2.3. Data Analysis

All of the variables were descriptively presented. Independent t-tests were used to examine the differences in continuous variables (e.g., patients’ DM knowledge, patients’ self-care behaviors, caregivers’ DM knowledge, and caregivers’ patient-care behaviors) between two groups of variables (e.g., male vs. female, married vs. unmarried). One-way ANOVA was used to examine differences in continuous variables for more than two groups of variables (e.g., body mass index (BMI) level, education level). Simple linear regression and Pearson’s correlation coefficients (r) were used to investigate the relationship of patients’ QoL with patients’ knowledge, caregivers’ knowledge, patients’ behaviors, and caregivers’ behaviors. Multiple linear regression was used to identify how caregivers’ knowledge and behaviors influenced patients’ QoL when adjusting for all potential factors that were found to be significant with patients’ QoL in the univariate analysis. Statistical significance was defined as *p* < 0.05. Statistical analyses were conducted using SPSS (SPSS Inc., Chicago, IL, USA) software for Windows.

## 3. Results

The personal and clinical characteristics of elderly diabetic patients are presented in Table 1. The majority of patients were female (70.3%), married (59.3%), and unemployed or retired (52.3%). Mean age and BMI of patients were 69.15 ± 6.93 years and 24.18 ± 4.36 kg/m^2^, respectively. Most of them had a primary level of education (65.8%) and financial difficulties (52.3%). Only 7.3% smoked, and 15.3% consumed alcohol. Regarding the household relationship or living arrangements, most lived with multilevel family members (54.8%), followed by living with only one spouse/son/daughter (36.4%), and only a few lived with relatives, such as a brother/sister or niece/nephew (8.8%). Regarding the diabetes clinical data, all had Type 2 diabetes, with a mean diagnosis duration of 10.93 ± 8.46 years. Almost all were treated with oral medications (74.9%); followed by both oral medications and insulin (22.0%); only lifestyle modification, such as dietary control, weight control, and exercise (2.0%); and only insulin therapy (1.1%). Comorbidity was found in 81.1%, with hypertension (63.3%) and dyslipidemia (27.6%) being the most common. One-fourth (26.3%) had a diabetes complication, 55.5% had eye complications (e.g., glaucoma, cataracts, or retinopathy), and 18.5% had nephropathy. The average ADL score of patients was 19.42 ± 1.92 points.

The personal characteristics and care information of family caregivers are presented in Table 2. The majority of caregivers were female (60.2%) and married (69.8%). Mean age and BMI of caregivers were 51.43 ± 14.57 years and 24.22 ± 4.23 kg/m^2^, respectively. Most of them had at least a secondary level of education (52.8%) and monthly family income of less than THB 5000 (43.2%). Only 11.0% smoked, and 32.5% consumed alcohol. The relationship of the caregiver to the patient included spouse (37.0%), son or daughter (38.7%), and relatives such as niece/nephew, sibling (24.3%). Regarding the care information, the average caring experience and time spent caring for the individual each day were 8.66 ± 5.45 years and 10.30 ± 8.29 h a day, respectively. More than half (59.6%) had a backup caregiver.

The results for diabetes knowledge and behavior for both groups, as well as patients’ QoL are presented in Table 3. Patients had an average DM knowledge score of 8.27 ± 3.93 points, while caregivers had a slightly higher DM knowledge score of 8.42 ± 3.79 points. In terms of behaviors, the mean SS of DSMQ score among patients was 7.14 ± 1.58 points, with the highest score in the GM subscale and the lowest score in the PA subscale. The mean score for patient-care behavior among caregivers was 7.40 ± 1.58 points, with the highest score in the DC subscale and the lowest score in the HU subscale. Most of the patients had a moderate level of QoL (55.9%), with an average score of 87.21 ± 13.01 points.

In the univariate analysis, patient factors that were significantly associated with their self-care behaviors were employment, household relationship, and comorbidity (*p* < 0.05) (Table 2). Patient factors significantly associated with QoL were age, BMI, education level, household relationship, and ADL (*p* < 0.05) (Table 2). No caregiver characteristics were significantly associated with patients’ self-care behaviors or QoL (Table 3). For the univariable analysis of the linear regression model, patients’ self-care behaviors were significantly associated with both patients’ (β = 0.344) and caregivers’ (β = 0.341) DM knowledge (Table 4). Each 1-point increase in DM knowledge among elderly patients or caregivers resulted in a 0.14-point increase in patients’ self-care behaviors. Because of the high positive correlation between DM knowledge of elderly patients and caregivers, only patients’ knowledge variable was included in the multivariable analysis to reduce collinearity. After controlling for relevant patient factors, such as employment, household relationship, and comorbidity, it was observed that DM knowledge of elderly patients was correlated with their self-care behaviors (β = 0.351, *p* < 0.05). These factors accounted for 15.3% of the variation in the self-care behavior score.

In terms of patients’ QoL, the univariable analysis revealed a statistically significant association with patients’ DM knowledge (β = 0.379), caregivers’ DM knowledge (β = 0.282), patients’ self-care behaviors (β = 0.363), and caregivers’ patient-care behaviors (β = 0.353) (Table 5). ADL and patients’ DM knowledge were excluded from the multivariable analysis to increase power and minimize multicollinearity. The results revealed that patients’ QoL score was positively correlated with caregivers’ DM knowledge (β = 0.169) and patient-care behaviors (β = 0.167) after adjusting for relevant patient factors, such as age, BMI, education level, household relationship, and patients’ self-care behaviors (R^2^ = 27.0%, *p* < 0.05). According to the model, each 1-point increase in caregivers’ DM knowledge and patient-care behaviors increased the QoL score by 0.58 and 1.38 points, respectively.

The correlation between patients’ and caregivers’ behaviors was classified by subscale and QoL among elderly diabetic patients (Table 6). A positive correlation was observed between patients’ QoL and self-care behaviors, including the subscales of GM (r = 0.266, *p* < 0.001), DC (r = 0.183, *p* = 0.001), PA (r = 0.287, *p* < 0.001), and HU (r = 0.335, *p* < 0.001). There was also a positive correlation between patients’ QoL and caregivers’ patient-care behaviors in the subscales of GM (r = 0.139, *p* = 0.009), DC (r = 0.327, *p* < 0.001), PA (r = 0.350, *p* < 0.001), and HU (r = 0.373, *p* < 0.001).

## 4. Discussion

The findings emphasize the link between DM knowledge and behaviors of both the patient and the caregiver and the patient’s QoL. The overall DM knowledge among patients in this study was consistent with a previous study of the Thai population, which found that the mean percentage of correct answers on the T-SDKS was 42.39% [27]. Similarly, according to the T-SDKS, most patients (≈90%) had no knowledge of specific DM items, such as glycosylated hemoglobin (HbA1C). However, the total knowledge score (8.3) was lower than that of DM patients in South Africa, who had scores of around 11.6 [32]. In addition, caregivers’ DM knowledge scores were comparable to those of the patients, with the lowest percentage of correct answers on the questions regarding HbA1C and testing for glucose when sick with the flu. This may imply that the two groups learned about DM from the same source or that they shared DM knowledge. These similarities in diabetes knowledge suggest that both elderly diabetic patients and their family caregivers should be educated and familiar with diabetes information, such as DM clinical data, treatment, and medications.

Regarding DM patients’ reported self-care behaviors, these were consistent with several studies from other countries, including Germany [28,33], Kuwait [4], Pakistan [14], Philippines [13], Saudi Arabia [34], Turkey [35], and the United States [36], which found DSMQ scores ranging from 4.4 to 7.8. The relatively high DSMQ score in this study (7.1) indicates that DM patients in this study had good self-care behaviors. Similar to earlier studies, the lowest score was for the PA subscale [14,33,35]. Additionally, the patient-care behavior score among family caregivers was highest for the DC subscale and lowest for the HU subscale. This indicates that family caregivers support or significantly contribute to proper food selection for promoting good health and nutrition status in elderly diabetes patients. Patients should be encouraged to practice good self-care, especially by engaging in physical activity. Finally, family caregivers should be empowered to promote good care behaviors, such as taking the patient to see a medical practitioner, monitoring blood sugar levels, and adhering to the doctor’s advice.

Almost all elderly DM patients had overall QoL scores that were moderate or high. This may be due to good self-management in this study sample group, which can have a direct impact on glycemic control and health status. The glycemic status of diabetic patients, such as an HbA1C test, should be collected in future investigations. Our results were similar to a previous study that found that DM patients’ QoL score was highest for the DAD facet and lowest for the SOP facet [18]. Most elderly people in our investigation were less concerned about death and accepted it. Regarding the low score for SOP, it may be that COVID-19 infection prevention and control measures limited their movement and interaction with others. Our findings are consistent with previous studies that have found that 54.3% of Thai patients with Type 2 DM had a good QoL [35]. A recent study found that 42.9% of Thai DM patients over the age of 60 had good QoL, which was lower compared to DM patients under the age of 60 [37]. According to the WHOQOL-OLD assessment, the majority of Thai older adults living in rural areas had a fair QoL [31], and those with diabetes had a lower QoL score than those without any disease [38]. In another study in Indonesia, the median overall QoL score of people over the age of 60 was 87 [39]. Older adults with a diagnosis of DM experienced poorer health-related QoL, especially regarding physical and mental health, compared to those without a diagnosis of DM [10,40]. These results emphasize that body deterioration and diabetes in the elderly have an impact on QoL.

The findings revealed that both patients’ and caregivers’ scores for DM knowledge were significantly correlated with the patients’ self-care behavior scores. In line with previous research, patients’ diabetes knowledge score was highly correlated with their self-care practice score in Lebanese urban adult patients with DM (r = 0.84) [15]. A study in Pakistan found a strong positive correlation between diabetes knowledge and the DSMQ SS among adults with diabetes (r = 0.63) [14]. Diabetes knowledge was significantly correlated with self-management in terms of blood glucose testing among people with Type 2 DM in Australia (r = 0.15) [16]. In addition, a knowledgeable caregiver may provide some informative advice to improve the patient’s health behaviors. Our findings indicate that family caregivers’ DM knowledge may help patients cope with suggested lifestyle changes.

Overall, DM knowledge and self-care behaviors were significantly correlated with QoL among patients. This is consistent with a previous study that observed a correlation between knowledge and QoL scores, particularly SAB, AUT, and INT facets, in elderly patients with DM [17]. A recent study found that DM patients who had a high level of knowledge regarding prevention and care had 3.3 times greater odds of having a good QoL than those who had a low level of knowledge [37]. Another study revealed that diabetes knowledge served as a moderator in the relationship between health literacy and glucose self-control [41]. Furthermore, disease knowledge and self-care practices were associated with DM patients’ glycemic control [14]. In our study, QoL was related to all subscales of self-care behavior, especially HU, PA, and GM. Komaratat et al. [18] found that self-care behaviors were positively correlated with QoL in patients with Type 2 DM, especially taking medicine, reducing the risk of complications, and controlling one’s emotions. Self-care nutrition behavior, self-management of blood glucose control, and self-medication behavior were predictors of the QoL [42]. Another study revealed that self-management in terms of diet, exercise, blood glucose testing, and foot care was associated with QoL among people with Type 2 diabetes [16]. We recommend an educational program to increase DM knowledge and improve self-care behaviors in elderly diabetic patients to increase their QoL.

In terms of family caregivers, DM knowledge and patient-care behaviors were significantly correlated with patients’ QoL. It is possible that the elderly may have more confidence in their caregiver’s ability to care of them if their caregivers have accurate and higher knowledge [43], which may directly impact their QoL. Another explanation for this finding could be that increased knowledge helps to improve patients’ behaviors and glycemic control, resulting in QoL improvements. We found that all subscales of caregivers’ behaviors, and especially HU, PA, and DC, were related to patients’ QoL. Family support includes encouraging communication, assisting with obtaining health services, reminding elderly patients to take blood sugar control medications as prescribed by a doctor on a regular basis, organizing diet, and checking blood sugar [20,21]. These may improve treatment and rehabilitation outcomes for patient care and have a direct impact on physical well-being and good QoL. A previous study found that elderly DM patients were happy and grateful to receive their family’s support [20]. This may reflect in their mental and emotional well-being. Theoretically, social support, such as informational and emotional supports, can improve a person’s morale and, subsequently, their health [43,44]. Another study found that social support, including emotional support, moral support, and appraisal support, was positively association with QoL [18]. Many studies have emphasized the importance of social support for changes in behaviors, health, and QoL [22]. Because of the complexity of diabetes management, some patients will be more reliant on their caregivers than others [45]. These findings suggest that family caregivers’ social support interventions may enhance DM management practices and QoL in elderly diabetes patients.

This study is limited by its cross-sectional nature, which means that causal relationships cannot be inferred. The COVID-19 outbreak situation may have affected the information obtained due to changes in activities and way of life. However, this study emphasized the importance of family caregivers’ knowledge and behaviors in promoting health and improving QoL in older diabetic patients, as well as providing evidence of the importance of implementing education and behavior change programs, not only for diabetes patients but also for their caregivers. Future research should include a physical examination, measurement of HbA1C, and qualitative methods to explore patients’ perceptions and opinions about patient-care behaviors of family caregivers on their QoL. More sampling sites in other provinces are suggested to generalize the findings to more of the elderly Thai DM population.

## 5. Conclusions

The findings demonstrated the influence of diabetes knowledge and behaviors among both elderly patients and their family caregivers on patients’ QoL. An intervention and measures should be provided for DM patients and their family caregivers simultaneously to improve their diabetes knowledge and behaviors, enhancing patients’ QoL, particularly their social well-being. Health care providers should focus more on advising and supporting family members to achieve long-term diabetes management goals.

## Figures and Tables

**Table 1:** Factors of elderly diabetic patients that related to their diabetes knowledge, self-care behavior, and QoL (*n* = 354).

Variable	(*n* = 354) *n* (%)	Diabetes Knowledge		Self-Care Behavior		Quality of Life	
		Mean ± SD	*p*-Value	Mean ± SD	*p*-Value	Mean ± SD	*p*-Value
**Sex**			0.017		0.858		0.961
Male	105 (29.7)	9.04 ± 4.08		7.16 ± 1.54		87.16 ± 13.50	
Female	249 (70.3)	7.95 ± 3.83		7.13 ± 1.60		87.24 ± 12.83	
**Age**			0.277		0.239		<0.001
60–69 years	210 (59.3)	8.21 ± 4.01		7.24 ± 1.60		88.91 ± 13.05	
70–79 years	108 (30.5)	8.66 ± 3.82		7.04 ± 1.56		86.87 ± 12.00	
≥80 years	36 (10.2)	7.47 ± 3.79		6.81 ± 1.50		78.33 ± 12.30	
**BMI**			0.554		0.123		0.006
<18.5 kg/m^2^	23 (6.5)	7.96 ± 4.81		6.89 ± 1.67		80.87 ± 8.78	
18.5–22.9 kg/ m^2^	126 (35.6)	7.96 ± 3.94		7.01 ± 1.56		86.60 ± 12.19	
23.0–24.9 kg/ m^2^	68 (19.2)	8.76 ± 3.86		6.95 ± 1.72		87.38 ± 13.14	
≥25.0 kg/ m^2^	137 (38.7)	8.37 ± 3.82		7.39 ± 1.48		88.77 ± 14.00	
**Education level**			0.348		0.582		<0.001
None	61 (17.3)	7.62 ± 4.03		6.95 ± 1.33		81.61 ± 10.17	
Primary school	233 (65.8)	8.37 ± 3.72		7.19 ± 1.62		87.92 ± 13.21	
Secondary school or higher	60 (16.9)	8.55 ± 4.59		7.12 ± 1.65		90.18 ± 13.34	
**Marital status**			0.467		0.698		0.961
Married	210 (59.3)	8.40 ± 4.07		7.17 ± 1.51		87.24 ± 13.03	
Unmarried	144 (40.7)	8.09 ± 3.72		7.10 ± 1.68		87.17 ± 13.04	
**Employment**			0.253		0.043		0.715
Employed	169 (47.7)	8.02 ± 3.99		7.31 ± 1.58		87.48 ± 12.62	
Unemployed	185 (52.3)	8.50 ± 3.88		6.97 ± 1.56		86.97 ± 13.39	
**Perceived financial status**			0.207		0.191		0.273
Sufficient	169 (47.7)	8.55 ± 4.05		7.02 ± 1.62		86.42 ± 13.50	
Insufficient	185 (52.3)	8.02 ± 3.82		7.24 ± 1.54		87.94 ± 12.54	
**Living arrangements**			0.578		0.037		<0.001
Multilevel family members	194 (54.8)	8.09 ± 3.87		7.26 ± 1.61		86.58 ± 13.69	
One spouse/son/daughter	129 (36.4)	8.58 ± 3.86		7.08 ± 1.51		89.50 ± 12.07	
Relatives	31 (8.8)	8.16 ± 4.65		6.60 ± 1.59		81.65 ± 10.44	
**Duration of DM**			0.262		0.845		0.257
≤5 years	111 (31.4)	8.77 ± 3.97		7.18 ± 1.74		88.68 ± 13.41	
6–9 years	71 (20.0)	8.14 ± 3.83		7.20 ± 1.53		87.62 ± 11.83	
≥10 years	172 (48.6)	8.01 ± 3.94		7.09 ± 1.50		86.10 ± 13.19	
**Smoking**			0.882		0.191		
Yes	26 (7.3)	8.38 ± 4.29		7.53 ± 1.51		90.62 ± 10.78	
No	328 (92.7)	8.27 ± 3.91		7.11 ± 1.58		86.95 ± 13.15	
**Alcohol consumption**			0.887		0.924		0.106
Yes	54 (15.3)	8.20 ± 3.94		7.16 ± 1.49		89.85 ± 12.17	
No	300 (84.7)	8.29 ± 3.94		7.14 ± 1.60		86.74 ± 13.12	
**Comorbidities**			0.124		0.041		0.425
Yes	287 (81.1)	8.12 ± 3.83		7.05 ± 1.53		86.95 ± 13.24	
No	67 (18.9)	8.94 ± 4.33		7.49 ± 1.75		88.36 ± 12.03	
**Complications**			0.101		0.727		0.643
Yes	93 (26.3)	7.70 ± 3.97		7.19 ± 1.60		86.68 ± 14.43	
No	261 (73.7)	8.48 ± 3.91		7.12 ± 1.58		87.41 ± 12.50	
**Activities in daily living**			0.887		0.218		<0.001
Dependent (≤12)	6 (1.7)	8.50 ± 3.02		6.35 ± 1.57		67.67 ± 15.44	
Independent (>12)	348 (98.3)	8.27 ± 3.95		7.16 ± 1.58		87.56 ± 12.73	

**Table 2:** Factors of family caregivers that related to elderly diabetic patients’ diabetes knowledge, self-care behavior and QoL (*n* = 354).

Variable	(*n* = 354) *n* (%)	Diabetes Knowledge		Self-Care Behaviors		Quality of Life	
		Mean ± SD	*p*-Value	Mean ± SD	*p*-Value	Mean ± SD	*p*-Value
**Sex**			0.062		0.059		0.627
Male	141 (39.8)	7.79 ± 3.90		7.34 ± 1.53		86.80 ± 12.55	
Female	213 (60.2)	8.59 ± 3.93		7.01 ± 1.60		87.49 ± 13.33	
**Age**			0.853		0.332		0.459
18–39 years	80 (22.6)	8.16 ± 3.32		7.15 ± 1.59		88.18 ± 11.63	
40–59 years	143 (40.4)	8.20 ± 3.77		7.00 ± 1.51		86.18 ± 13.41	
≥60 years	131 (37.0)	8.43 ± 4.44		7.29 ± 1.64		87.76 ± 13.39	
**BMI**			0.271		0.577		0.558
<18.5 kg/m^2^	26 (7.3)	7.35 ± 3.76		6.92 ± 1.53		84.81 ± 13.15	
18.5–22.9 kg/m^2^	115 (32.5)	7.90 ± 4.03		7.13 ± 1.69		86.41 ± 12.81	
23.0–24.9 kg/m^2^	68 (19.2)	8.65 ± 3.91		7.36 ± 1.60		88.30 ± 13.89	
≥25.0 kg/m^2^	145 (41.0)	8.57 ± 3.88		7.09 ± 1.50		87.77 ± 12.77	
**Education level**			0.852		0.833		0.423
None	14 (4.0)	8.86 ± 3.25		7.05 ± 1.61		88.93 ± 17.00	
Primary school	153 (43.2)	8.24 ± 4.00		7.09 ± 1.67		88.09 ± 13.63	
Secondary school or higher	187 (52.8)	8.26 ± 3.93		7.19 ± 1.50		86.37 ± 12.15	
**Marital status**			0.531		0.185		0.564
Married	247 (69.8)	8.36 ± 3.81		7.21 ± 1.55		86.95 ± 13.40	
Unmarried	107 (30.2)	8.07 ± 4.22		6.98 ± 1.63		87.82 ± 12.12	
**Monthly family income**			0.855		0.419		0.268
<5000 THB	153 (43.2)	8.39 ± 3.97		7.24 ± 1.63		88.29 ± 13.29	
5000-10,000 THB	95 (26.8)	8.26 ± 3.92		7.17 ± 1.54		85.53 ± 12.98	
≥10,001 THB	106 (30.0)	8.11 ± 3.92		6.98 ± 1.54		87.18 ± 12.59	
**Smoking**			0.657		0.195		0.056
Yes	39 (11.0)	8.54 ± 3.83		6.83 ± 1.64		83.46 ± 12.33	
No	315 (89.0)	8.24 ± 3.95		7.18 ± 1.56		87.68 ± 13.04	
**Alcohol consumption**			0.989		0.879		0.234
Yes	115 (32.5)	8.28 ± 4.03		7.12 ± 1.50		86.03 ± 1267	
No	239 (67.5)	8.27 ± 3.89		7.15 ± 1.62		87.79 ± 13.16	
**Caregiver relationship to patient**			0.703		0.448		0.858
Spouse	131 (37.0)	8.35 ± 4.48		7.28 ± 1.60		87.16 ± 13.33	
Son or daughter	137 (38.7)	8.39 ± 3.50		7.08 ± 1.48		87.63 ± 12.62	
Relatives	86 (24.3)	7.97 ± 3.70		7.03 ± 1.70		86.64 ± 13.27	
**Years of caring experience**			0.695		0.598		0.435
<5 years	166 (46.9)	8.19 ± 3.90		7.19 ± 1.54		86.64 ± 11.96	
≥5 years	188 (53.1)	8.35 ± 3.97		7.10 ± 1.61		87.72 ± 13.89	
**Time spent caring**			0.107		0.855		0.892
≤7 h/day	164 (46.3)	8.32 ± 4.25		7.10 ± 1.67		86.87 ± 12.37	
8–16 h/day	102 (28.8)	8.79 ± 3.51		7.14 ± 1.41		87.63 ± 13.11	
≥17 h/day	88 (24.9)	7.59 ± 3.71		7.22 ± 1.60		87.21 ± 14.16	
**Having secondary caregiver**			0.257		0.786		0.773
Yes	211 (59.6)	8.47 ± 3.83		7.12 ± 1.55		87.38 ± 13.84	
No	143 (40.4)	7.99 ± 4.07		7.16 ± 1.62		86.97 ± 11.73	

**Table 3:** Diabetes knowledge and behavior for both groups, and patients’ QoL.

Variable	*n* (%)	Mean ± SD	Min–Max
**Patients’ diabetes knowledge**			
Total score		8.27 ± 3.93	0–20
Percentile:			
76–100% correct	11 (3.1)		
51–75% correct	90 (25.4)		
26–50% correct	160 (45.2)		
0–25% correct	93 (26.3)		
**Patients’ self-care behaviors**			
Total DSMQ (sum-scale; SS)		7.14 ± 1.58	2.50–10.00
Subscale:			
Glucose management (GM)		7.60 ± 2.15	0.67–10.00
Dietary control (DC)		7.17 ± 1.82	1.70–10.00
Physical activity (PA)		6.34 ± 2.51	0.00–10.00
Health-care use (HU)		7.23 ± 1.98	1.10–10.00
**Patients’ QOL**			
Total QOL		87.21 ± 13.01	37–120
Low level (≤55)	2 (0.6)		
Moderate level (56–88)	198 (55.9)		
High level (≥89)	154 (43.5)		
Facet:			
Sensory functioning (SAB)		14.72 ± 3.33	5–20
Autonomy (AUT)		13.65 ± 3.09	4–20
Past present and future activities (PFF)		14.39 ± 2.64	6–20
Social participation (SOP)		13.62 ± 3.04	4–20
Death and dying (DAD)		16.53 ± 3.27	6–20
Intimacy (INT)		14.30 ± 2.56	4–20
**Caregivers’ diabetes knowledge**			
Total score		8.42 ± 3.79	0–20
Percentile:			
76–100% correct	12 (3.4)		
51–75% correct	91 (25.7)		
26–50% correct	168 (47.5)		
0–25% correct	83 (23.4)		
**Caregivers’ patient-care behaviors**			
Total DSMQ (sum-scale; SS)		7.40 ± 1.58	3.33–10.00
Subscale:			
Glucose management (GM)		7.34 ± 2.08	1.33–10.00
Dietary control (DC)		7.84 ± 1.67	2.50–10.00
Physical activity (PA)		7.27 ± 2.34	0.00–10.00
Health-care use (HU)		7.22 ± 1.82	2.22–10.00

**Table 4:** Relationship between diabetes knowledge for both groups and patients’ self-care behaviors.

Variable	B	SE	Beta	*p*-Value	R Square
**Univariable**					
(Constant)	6.00	0.18		<0.001 *	11.9%
Patients’ diabetes knowledge (score)	0.14	0.02	0.344	<0.001 *	
(Constant)	5.94	0.19		<0.001 *	11.7%
Caregivers’ diabetes knowledge (score)	0.14	0.02	0.341	<0.001 *	
**Multivariable**					
(Constant)	6.40	0.41		<0.001 *	15.3%
Employment (unemployed)	−0.36	0.16	−0.114	0.028 *	
Comorbidities (no)	0.25	0.21	0.061	0.233	
Living arrangements:					
Multilevel family members	0.61	0.29	0.193	0.033 *	
One spouse/son/daughter	0.32	0.30	0.097	0.287	
Relatives	Ref.				
Patients’ diabetes knowledge (score)	0.14	0.02	0.351	<0.001 *	

B = Unstandardized Regression Coefficient; SE = Standard Error; Beta = Standardized Regression Coefficients. * Significance at the 0.05 level.

**Table 5:** Relationship between diabetes knowledge and behavior for both groups and patients’ QoL.

Variable	B	SE	Beta	*p*-Value	R Square
**Univariable**					
(Constant)	76.85	1.50		<0.001 *	14.3%
Patients’ diabetes knowledge (score)	1.25	0.16	0.379	<0.001 *	
(Constant)	65.84	2.99		<0.001 *	13.2%
Patients’ self-care behaviors (score)	3.00	0.41	0.363	<0.001 *	
(Constant)	79.07	1.62		<0.001 *	7.9%
Caregivers’ diabetes knowledge (score)	0.97	0.18	0.282	<0.001 *	
(Constant)	65.62	3.12		<0.001 *	12.4%
Caregivers’ patient-care behaviors (score)	2.92	0.41	0.353	<0.001 *	
**Multivariable**					
(Constant)	76.93	8.93		<0.001 *	27.0%
Patients’ ages (years)	−0.34	0.09	−0.183	<0.001 *	
Patients’ BMI (kg/m^2^)	0.06	0.15	0.020	0.680	
Patients’ education level:					
None	Ref.				
Primary school	3.35	1.71	0.122	0.051	
Secondary school or higher	5.84	2.12	0.169	0.006	
Living arrangements:					
Multilevel family members	2.00	2.21	0.077	0.365	
One spouse/son/daughter	4.99	2.28	0.185	0.030 *	
Relatives	Ref.				
Patients’ self-care behaviors (score)	1.58	0.48	0.192	0.001 *	
Caregivers’ diabetes knowledge (score)	0.58	0.17	0.169	0.001 *	
Caregivers’ patient-care behaviors (score)	1.38	0.47	0.167	0.004 *	

B = Unstandardized Regression Coefficient; SE = Standard Error; Beta = Standardized Regression Coefficients. * Significance at the 0.05 level.

**Table 6:** Pearson correlation coefficients (r) between patients’ and caregivers’ behaviors classified by subscale and patients’ QoL.

Variable	SS	GM	DC	PA	HU	QoL
**Patients’ self-care behaviors and QoL**						
Sum-scale (SS)	1					
Glucose management (GM)	0.772 *	1				
Dietary control (DC)	0.769 *	0.421 *	1			
Physical activity (PA)	0.680 *	0.243 *	0.489 *	1		
Health-care use (HU)	0.703 *	0.452 *	0.420 *	0.331 *	1	
Patients’ QoL	0.363 *	0.266 *	0.183 *	0.287 *	0.335 *	1
**Caregivers’ patient-care behaviors and patients’ QoL**						
Sum-scale (SS)	1					
Glucose management (GM)	0.766 *	1				
Dietary control (DC)	0.819 *	0.480 *	1			
Physical activity (PA)	0.812 *	0.402 *	0.648 *	1		
Health-care use (HU)	0.733 *	0.376 *	0.524 *	0.553 *	1	
Patients’ QoL	0.353 *	0.139 *	0.327 *	0.350 *	0.373 *	1

* Significance at the 0.01 level.

## Data Availability

Not applicable.

## References

[1] Baena-Díez J.M., Peñafiel J., Subirana I., Ramos R., Elosua R., Marín-Ibañez A., Guembe M.J., Rigo F., Tormo-Díaz M.J., Moreno-Iribas C. (2016). Risk of Cause-Specific Death in Individuals With Diabetes: A Competing Risks Analysis. Diabetes Care.

[2] Spasić A., Catic Djordjevic A., Stefanovic N., Tatjana C. (2014). Quality of Life in Type 2 Diabetic Patients. Acta Fac. Med. Naissensis.

[3] Ogurtsova K., Da Rocha Fernandes J.D., Huang Y., Linnenkamp U., Guariguata L., Cho N.H., Cavan D., Shaw J.E., Makaroff L.E. (2017). IDF Diabetes Atlas: Global estimates for the prevalence of diabetes for 2015 and 2040. Diabetes Res. Clin. Pract..

[4] Al-Khaledi M., Al-Dousari H., Al-Dhufairi S., Al-Mousawi T., Al-Azemi R., Al-Azimi F., Hanan (2018). Diabetes Self-Management: A Key to Better Health-Related Quality of Life in Patients with Diabetes. Med. Princ. Pract..

[5] John R., Pise S., Chaudhari L., Deshpande P. (2019). Evaluation of quality of life in type 2 diabetes mellitus patients using quality of life instrument for indian diabetic patients: A cross-sectional study. J. Mid-Life Health.

[6] Sun H., Saeedi P., Karuranga S., Pinkepank M., Ogurtsova K., Duncan B.B., Stein C., Basit A., Chan J.C.N., Mbanya J.C. (2022). IDF Diabetes Atlas: Global, regional and country-level diabetes prevalence estimates for 2021 and projections for 2045. Diabetes Res. Clin. Pract..

[7] Aekplakorn W., Chariyalertsak S., Kessomboon P., Sangthong R., Inthawong R., Putwatana P., Taneepanichskul S. (2021). The Sixth National Health Examination Survey 2020–21.

[8] Bureau of Non Communicable Disease (2022). The Number and Mortalities Rate Report of Non Communicable Disease 2016–2021.

[9] Lee M.K., Oh J. (2020). Health-Related Quality of Life in Older Adults: Its Association with Health Literacy, Self-Efficacy, Social Support, and Health-Promoting Behavior. Healthcare.

[10] Shamshirgaran S.M., Stephens C., Alpass F., Aminisani N. (2020). Longitudinal assessment of the health-related quality of life among older people with diabetes: Results of a nationwide study in New Zealand. BMC Endocr. Disord..

[11] Singh H., Bradley C. (2006). Quality of life in diabetes. Int. J. Diabetes Dev. Ctries..

[12] Rubin R.R., Peyrot M. (1999). Quality of life and diabetes. Diabetes/Metab. Res. Rev..

[13] Totesora D., Ramos-Rivera M.I., Villegas-Florencio M.Q., Reyes-Sia P.N. (2019). Association of Diabetes-related Emotional Distress with Diabetes Self-care and Glycemic Control among Adult Patients with Type 2 Diabetes at a Tertiary Hospital in Manila, Philippines. J. ASEAN Fed. Endocr. Soc..

[14] Bukhsh A., Khan T.M., Sarfraz Nawaz M., Sajjad Ahmed H., Chan K.G., Goh B.-H. (2019). Association of diabetes knowledge with glycemic control and self-care practices among Pakistani people with type 2 diabetes mellitus. Diabetes Metab. Syndr. Obes. Targets Ther..

[15] Karaoui L.R., Deeb M.E., Nasser L., Hallit S. (2018). Knowledge and practice of patients with diabetes mellitus in Lebanon: A cross-sectional study. BMC Public Health.

[16] Kueh Y.C., Morris T., Borkoles E., Shee H. (2015). Modelling of diabetes knowledge, attitudes, self-management, and quality of life: A cross-sectional study with an Australian sample. Health Qual. Life Outcomes.

[17] Sousa M.C.d., Dias F.A., Nascimento J.S., Tavares D.M.D.S. (2016). Correlation of quality of life with the knowledge and attitude of diabetic elderly. Investig. Educ. Enfermería.

[18] Komaratat C., Auemaneekul N., Kittipichai W. (2020). Quality of life for type II diabetes mellitus patients in a suburban tertiary hospital in Thailand. J. Health Res..

[19] Babazadeh T., Dianatinasab M., Daemi A., Nikbakht H.A., Moradi F., Ghaffari-Fam S. (2017). Association of Self-Care Behaviors and Quality of Life among Patients with Type 2 Diabetes Mellitus: Chaldoran County, Iran. Diabetes Metab. J..

[20] Kristianingrum N.D., Wiarsih W., Nursasi A.Y. (2018). Perceived family support among older persons in diabetes mellitus self-management. BMC Geriatr..

[21] Bennich B.B., Røder M.E., Overgaard D., Egerod I., Munch L., Knop F.K., Vilsbøll T., Konradsen H. (2017). Supportive and non-supportive interactions in families with a type 2 diabetes patient: An integrative review. Diabetol. Metab. Syndr..

[22] Larocca M.A., Scogin F.R. (2015). The Effect of Social Support on Quality of Life in Older Adults Receiving Cognitive Behavioral Therapy. Clin. Gerontol..

[23] Mahoney F.I., Barthel D.W. (1965). Functional Evaluation: The Barthel Index. Md. State Med. J..

[24] Jitapunkul S. (1994). Disability: A problem of the elderly. Chula Med. J..

[25] Jitapunkul S., Kunanusont C., Phoolcharoen W., Suriyawongpaisal P., Ebrahim S. (2003). Disability-free life expectancy of elderly people in a population undergoing demographic and epidemiologic transition. Age Ageing.

[26] Collins G.S., Mughal S., Barnett A.H., Fitzgerald J., Lloyd C.E. (2010). Modification and validation of the Revised Diabetes Knowledge Scale. Diabet. Med..

[27] Khunkaew S., Fernandez R., Sim J. (2018). Linguistic and Psychometric Validation of the Thai Version of Simplified Diabetes Knowledge Scale: A Measure of Knowledge of Diabetes in a Thai Population. SAGE Open Nurs..

[28] Schmitt A., Gahr A., Hermanns N., Kulzer B., Huber J., Haak T. (2013). The Diabetes Self-Management Questionnaire (DSMQ): Development and evaluation of an instrument to assess diabetes self-care activities associated with glycaemic control. Health Qual. Life Outcomes.

[29] Thojampa S., Mawn B. (2017). Psychometric evaluation of the Thai translation of the Diabetes Self-management Questionnaire in type 2 diabetes. Int. J. Nurs. Sci..

[30] World Health Organization (2004). Whoqol-Old Manual.

[31] Hongthong D., Somrongthong R., Ward P. (2015). Factors Influencing the Quality of Life (Qol) among Thai Older People in a Rural Area of Thailand. Iran. J. Public Health.

[32] Muchiri J.W., Gericke G.J., Rheeder P. (2021). Effectiveness of an adapted diabetes nutrition education program on clinical status, dietary behaviors and behavior mediators in adults with type 2 diabetes: A randomized controlled trial. J. Diabetes Metab. Disord..

[33] Brenk-Franz K., Strauss B., Tiesler F., Fleischhauer C., Ciechanowski P., Schneider N., Gensichen J. (2015). The Influence of Adult Attachment on Patient Self-Management in Primary Care—The Need for a Personalized Approach and Patient-Centred Care. PLoS ONE.

[34] Alodhayani A., Almutairi K.M., Vinluan J.M., Almigbal T.H., Alonazi W.B., Ali Batais M., Mohammed Alnassar M. (2021). Association between self-care management practices and glycemic control of patients with type 2 diabetes mellitus in Saud Arabia: A cross-sectional study. Saudi J. Biol. Sci..

[35] Utli H., Vural Doğru B. (2021). The effect of the COVID-19 pandemic on self-management in patients with type 2 diabetics. Prim. Care Diabetes.

[36] Jia J., Jenkins A.J., Quintiliani L.M., Truong V., Lasser K.E. (2022). Resilience and diabetes self-management among African-American men receiving primary care at an urban safety-net hospital: A cross-sectional survey. Ethn. Health.

[37] Tamornpark R., Utsaha S., Apidechkul T., Panklang D., Yeemard F., Srichan P. (2022). Quality of life and factors associated with a good quality of life among diabetes mellitus patients in northern Thailand. Health Qual. Life Outcomes.

[38] Ong-Artborirak P., Seangpraw K. (2019). Association Between Self-Care Behaviors and Quality of Life Among Elderly Minority Groups on the Border of Thailand. J. Multidiscip. Healthc..

[39] Gondodiputro S., Wiwaha G., Lionthina M., Sunjaya D.K. (2021). Reliability and validity of the Indonesian version of the World Health Organization quality of life-old (WHOQOL-OLD): A Rasch modeling. Med. J. Indones..

[40] Wändell P.E., Tovi J. (2000). The quality of life of elderly diabetic patients. J. Diabetes Its Complicat..

[41] Van der Heide I., Uiters E., Rademakers J., Struijs J.N., Schuit A.J., Baan C.A. (2014). Associations among health literacy, diabetes knowledge, and self-management behavior in adults with diabetes: Results of a dutch cross-sectional study. J. Health Commun..

[42] Chantriyawong S. (2018). Developing a guide to promoting the quality of life for the elderly for families. J. Community Dev. Qual. Life.

[43] Schaefer C., Coyne J.C., Lazarus R.S. (1981). The health-related functions of social support. J. Behav. Med..

[44] Berkman L.F. (2000). Social support, social networks, social cohesion and health. Soc. Work Health Care.

[45] Alnaim L., Altuwaym R.A., Aldehan S.M., Alquraishi N.M. (2021). Assessment of knowledge among caregivers of diabetic patients in insulin dosage regimen and administration. Saudi Pharm. J..

